# Modulation of Performance, Plasma Constituents, Small Intestinal Morphology, and Cecum Microbiota in Growing Geese by Dietary Citric Acid Supplementation

**DOI:** 10.3390/ani14050660

**Published:** 2024-02-20

**Authors:** Yongkang Zhang, Jiajia Xue, Ying Chen, Xiaofeng Huang, Zuolan Liu, Hang Zhong, Qun Xie, Yi Luo, Qigui Wang, Chao Wang

**Affiliations:** 1 Poultry Science Institute, Chongqing Academy of Animal Sciences, Rongchang, Chongqing 402460, China; 17358362011@163.com (Y.Z.); xuejiajia25@163.com (J.X.); chenying.cq@163.com (Y.C.); hxf2007@yeah.net (X.H.); liuzuolan9229@163.com (Z.L.); hangzhongcqaas@163.com (H.Z.); xiequn20221208@163.com (Q.X.); homleestar@163.com (Y.L.); wangqigui@hotmail.com (Q.W.); 2 Scientific Observation and Experiment Station of Livestock Equipment Engineering in Southwest, Ministry of Agriculture, Rongchang, Chongqing 402460, China

**Keywords:** citric acid, performance, plasma constituents, small intestinal morphology, cecum microbiota

## Abstract

**Simple Summary:**

Goose meat is an ideal nutrition source with high protein, low fat, and low cholesterol, and dietary acidifiers supplementation are considered an efficient nutritional regulation approach to promote poultry industry development. Hence, this study was conducted to evaluate the benefits of citric acid supplementation in growing geese. The specific results found that citric acid supplementation showed a significant growth-promoting function, and benefited the antioxidant capacity and cecum microbiota of the geese. An inclusion level of 3.2% CA in growing geese diets is recommended based on this study.

**Abstract:**

To investigate the efficiency and optimum inclusion level of CA in growing geese diets on performance, plasma constituents, and intestinal health, 240 healthy female geese at the age of 28d were randomly allotted six treatment diets incorporated with 0, 0.8, 1.6, 2.4, 3.2, and 4% CA. Each treatment group consisted of five replicates and eight birds per replicate. The findings demonstrated that 3.2% CA supplementation resulted in improved growth performance (ADG, ADFI, and FBW) (*p* = 0.001), and geese who received CA also showed lower body fat contents (*p* < 0.05) than the control group. Moreover, geese from the 2.4% and 3.2% CA group had the highest plasma glutathione peroxidase and insulin-like growth factor 1 levels compared to the other groups (*p* < 0.05). A microbial diversity analysis of the cecum conducted by 16S rDNA sequencing revealed that 3.2% CA supplementation showed a significantly higher abundance of beneficial bacteria (*Muribaculaceae*, *CHKCI001*, *Erysipelotricha-ceae_UCG_003*, and *UCG_009*) (*p* < 0.05) and a lower abundance of harmful bacteria (*Atopobiaceae*, *Streptococcus*, *Acinetobacter*, *Pseudomonas*, and *Alistipes*) (*p* < 0.10). Collectively, our results revealed that dietary supplementation with 3.2% CA had several benefits on the performance and physiological health of growing geese by promoting nutrients metabolism, improving antioxidant capacity, and modulating cecum microbiota.

## 1. Introduction

Accumulated evidence supports the favorable effects of acidifiers in nutrient digestion and absorption, immune, antibacteria, and antioxidant aspects by lowering gastrointestinal pH and modulating gut microbiota [1,2,3]. Citric acid (CA) is a weak organic acid which is widely distributed in the fruits of plants and tissues of animals. As an organic acidifier and key component of the tricarboxylic acid (TCA) cycle, CA is more efficient, safe, and environmentally friendly than other acidifiers, such as malic, fumaric [3], and tartaric acid [4].

Studies have shown that adding CA to animal diets could stimulate appetite, lower the pH value of the gastrointestinal tract (GIT), activate digestive enzymes, and promote the proliferation of probiotics [5,6,7,8]. The increased probiotics may elevate the secretion of intestinal lactic acid and short-chain fatty acids (SCFAs), which have been confirmed to be conducive to the renewal and development of epithelial cells [9]. In addition, as a key component of the TCA cycle, CA could also directly synthesize ATP under stress, reducing the damage caused by stress to the body. Recent reports about CA supplementation in poultry diets have demonstrated that CA could promote the growth, immune status, intestinal structure, and barrier function of broilers [1,10], quails [6], and ducks [11]; however, reports on the effects on the physical and chemical properties of the diets and gut microbiome of geese are very rare, especially in their growing period.

Poultry is one of the cheapest sources of animal protein, of which goose meat is also an ideal nutritious and healthy food with high protein, low fat, and low cholesterol [12]. China is the largest producer and consumer of goose meat in the world, with 466 million geese used for meat production in 2022 [13]. Previous research by our team has demonstrated that the optimal level of CA in the gosling diets should be 1% [14]. Based on the above research, we further investigated the changes in feeding diets (pH value and acid-binding capacity) and blood hormone levels in growing geese with the increase in CA levels. Moreover, in the present study, we used 16s rDNA sequencing technology instead of the spread plate count method for a more in-depth analysis of the cecum microbiota composition. In conclusion, we hypothesized that the dietary supplementation of CA is also expected to have favorable effects on the growing geese. Hence, this research was performed to further evaluate the effects of dietary CA supplementation at different levels (0.8%, 1.6%, 2.4%, 3.2%, and 4%) on the performance, plasma constituents, small intestinal development, and cecum microbiota of growing Sichuan white geese.

## 2. Materials and Methods

### 2.1. Birds and Experimental Design

Initially, four-hundred 1 d old healthy female Sichuan white goslings were reared in the same management conditions until 28 d of age. Based on our previous findings and considering that growing geese might have a higher acid-resistant capacity than goslings, we set the maximum addition level of citric acid at 4%. Then, two hundred forty geese were selected and randomly divided into 6 treatment diets incorporated with 0, 0.8, 1.6, 2.4, 3.2, and 4% of CA, respectively. Each treatment group consisted of 5 replicates and 8 birds per replicate. The ingredients and nutrient levels of the basal diet are presented in Table 1, and the chemical analytical procedures were conducted according to the methods described in the AOAC [15]. The nitrogen content was determined using a continuous flow analytical system (AA3, Bran & Luebbe, Norderstedt, Germany), and the content of crude protein (CP) was obtained by multiplying the nitrogen content by 6.25 (Method 990.03). The ether extract (EE) was analyzed using an automatic fat extractor (SOX416, Gerhardt, Königswinter, Germany) according to Method 920.39, and the amino acid content was examined using an auto amino acid analyzer (L8900, Hitachi, Japan). All geese had ad libitum diets and clean water throughout the study. CA (anhydrous form, 99.5% purity) was obtained from Shandong Ensign Industrial Co. Ltd., Weifang, China. All treatment diets were pelleted, and no antibiotics were added.

### 2.2. Dietary pH Value and Acid-Binding Capacity

Each treatment diet was separately collected and stored at 4 °C. Accurately weighed 10 g dietary samples were dissolved in 90 mL pure water, then placed them in a constant-temperature shaker at 25 °C for 2 h. After that, the pH value of each sample was measured with a precision pH meter (Mettler Toledo Inc., Shanghai, China). The acid-binding capacity of each treatment diet was measured according to Bolduan et al. [16]. Concisely, 100 g of the dietary samples was weighed and dissolved in 200 mL pure water, heated to 37 °C in a constant-temperature water bath, then titrated with 1 mol/L hydrochloric acid until pH = 4. The milliliters consumed of hydrochloric acid was recorded, which is the acid-binding capacity of the treatment diets.

### 2.3. Growth Performance and Carcass Traits

The average daily gain (ADG) was calculated from the initial BW (IBW) and final BW (FBW), which were, respectively, weighted on a pen basis on d 28 and 70 after 8 h of fasting. Feed consumption was measured to determine the average daily feed intake (ADFI) on a pen basis, then the feed/gain ratio (F/G) was calculated. Dead birds were weighted accurately to correct the ADFI and F/G. Then, five geese from every group (near the average body weight of each replicate) were selected for slaughter (a total of 30). All carcass parts were weighted, and their percentage rates were calculated based on their slaughter weight.

### 2.4. Plasma Constituents

Blood samples were obtained from the chosen 30 birds for slaughter by puncturing their left-wing vein, and about 5 mL samples were collected into a vacuum tube with an anticoagulant. The samples were then processed through centrifugation at room temperature to separate the plasma (3000× *g*, 20 min) and then stored at −70 °C for further analysis. Plasma metabolites including uric acid (UA), urea (Urea), creatinine (CREA), the total protein (TP), globulin (GLO), and albumin (ALB) were determined with a fully automatic biochemical analyzer (AU680, Beckman Coulter, Tokyo, Japan). The analysis of growth hormones (GHs), insulin-like growth factor-1 (IGF-1), and immunoglobulins (IgA and IgG), as well as antioxidant indicators, including total antioxidant capacity (T-AOC), catalase (CAT), glutathione peroxidase (GSH-Px), malondialdehyde (MDA), and superoxide dismutase (SOD) were performed using corresponding commercial ELISA kits. All the relevant kits were purchased from Nanjing Jiancheng Bioengineering Institute (Nanjing, China).

### 2.5. Small Intestinal pH and Morphology

The small intestine segments (duodenum, jejunum, and ileum) were excised according to Liu et al. [17]. Then, the digesta of each small intestinal segment were separately collected to measure the pH of each segment with a pH meter conferring with the procedures of Chaveerach et al. [18]. Furthermore, approximately a 1 cm tissue sample was collected from the midpoints of the small intestine segments and then washed with 0.1 M phosphate-buffered saline. After that, these tissues were preserved by fixing them in 10% neutral buffered formalin for morphological analysis. These fixed tissues were subsequently dehydrated, processed, embedded in paraffin; cut into 5 μm thick slices; and stained with hematoxylin and eosin. 10 intact and well-oriented slices were chosen to determine the villus height (VH) and crypt depth (CD) of each small intestinal section with a digital camera microscope (BA400 Digital) under the Motic Advanced 3.2 digital image analysis system, and the ratio of villus height to crypt depth (VH/CD) was subsequently calculated.

### 2.6. Cecal Microbiota Analysis

Microbiota DNA of the cecal was extracted using a corresponding DNA Kit (S96, Tiangen Biotech, Beijing, China) from the control and the 3.2% CA groups, considering that the 3.2% CA group showed the best performance data. The 16S rDNA of distinct regions (V3 to V4) was selected for PCR amplification by specific primers (338F and 806R). Biomarker Technologies Co. Ltd. (Beijing, China) performed purified amplicon sequencing on an Illumina Miseq PE250 platform. The output data of the high-throughput sequencing were analyzed using the BMKCloud platform (www.biocloud.net).

### 2.7. Statistical Analysis

Data were tested by one-way ANOVA and Tukey’s range test to determine any differences using Software SPSS (version 26.0, Statistics, IBM Corporation, Chicago, IL, USA), where each pen was considered as an experimental unit. Statistically significant differences in cecum microbiota diversity were determined using the nonparametric Kruskal–Wallis test because microbiota data are not normally distributed. *p* < 0.05 was used to determine statistical significance, and 0.05 < *p* < 0.10 was considered a tendency.

## 3. Results

### 3.1. Dietary pH Value and Acid-Binding Capacity

As shown in Table 2, the dietary pH value and acid-binding capacity were significantly decreased by the increasing CA level (*p* < 0.01).

### 3.2. Growth Performance

As presented in Table 3, the geese from the 3.2% CA group showed a significantly higher ADG (*p* < 0.01) and ADFI (*p* < 0.01) and heavier FBW (*p* < 0.01) compared with the geese fed a basic diet. Interestingly, these indicators did not increase further with the increase in the CA addition level (up to 4%).

### 3.3. Carcass Traits

As shown in Table 4, geese from the 1.6% and 3.2% CA group had higher a thigh muscle percentage (TMP, *p* = 0.045) than the geese fed a basic diet. In addition, geese who received CA also showed lower body fat contents (SFP and AFP, *p*< 0.05) than the control group. There were no statistical differences observed in the dressing percentage (DP), eviscerated percentage (EP), breast muscle percentage (BMP), heart percentage (HP), liver percentage (LP), and proventriculus–gizzard percentage (PGP) (*p* > 0.05).

### 3.4. Plasma Constituents

Variance analysis revealed that the 2.4% CA group showed significantly higher plasma TP and GLO levels than the 4% CA group (*p* < 0.05, Table 5). Compared with the control group, the Urea of the 0.8% and 3.2% CA groups was significantly decreased (*p* < 0.05), and the UA of the 1.6% CA group was also significantly decreased (*p* < 0.05). As for the immune and antioxidant indices, CA addition only significantly (*p* = 0.019) affected the GSH-Px activity, and the 3.2% CA group showed the highest activity of GSH-Px, while no statistical differences were observed for the remaining parameters. Moreover, geese fed the 3.2% CA diet had significantly higher levels of IGF-1 (*p* = 0.039).

### 3.5. Small Intestinal pH and Morphology

According to Table 6, only the jejunum content pH showed a tendency to be reduced by CA addition (*p* = 0.087). Moreover, the duodenum VH/CD (*p* = 0.087) and jejunum VH/CD (*p* = 0.080) also tended to be increased by CA supplementation, and geese from the 3.2% CA group showed the highest VH/CD among all treatments. No significant effects were observed in the VH and CD of any small intestinal segment (*p* > 0.05).

### 3.6. Cecum Microbiota Diversity and Composition

Considering the important roles of cecum microbiota in the host’s nutrient metabolism, immune regulation, and physiological homeostasis [9], we speculate that the cecum’s microbiota also contribute to the growth and health in geese with CA supplementation. Geese fed 3.2% CA supplemented diets showed the best growth performance, carcass traits, nutritional and metabolic status, antioxidant capacity, and intestinal morphology, so we further investigated the effect of 3.2% CA supplementation in diets on gut microbiota.

**Figure 1:**
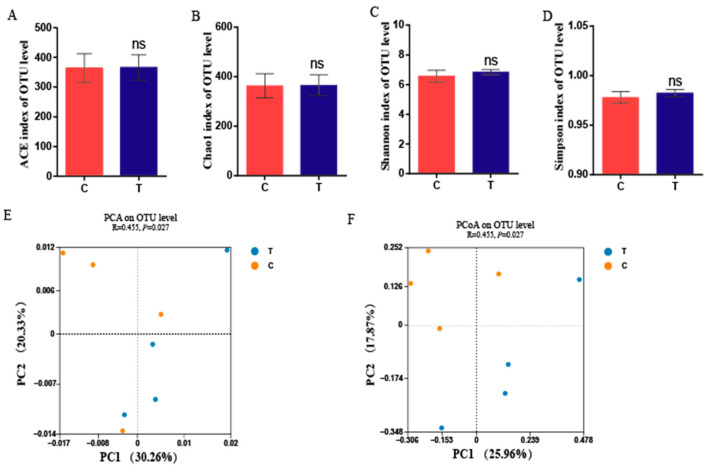
Alpha and beta diversity of cecal microbiota between the control group (**C**) and 3.2% CA group (T). (**A**–**D**) Alpha diversity index. (**E**) PCA plot of the cecal microbiota composition at the OUT level. (**F**) PCoA analysis based on Bray–Curtis distance at OUT level.

As shown in Figure 1A–D, geese from the 3.2% CA group showed no significant difference in Chao1, ACE, and Simpson and Shannon indexes compared with the control group (*p* > 0.05). The principal coordinate analysis (PCoA) and principal component analysis (PCA) plots showed that the samples were clustered together according to the different groups at the OTU level (*p* = 0.027, Figure 1E,F), indicating that the structure of geese cecum microbes was significantly changed after feeding with 3.2% CA.

**Figure 2:**
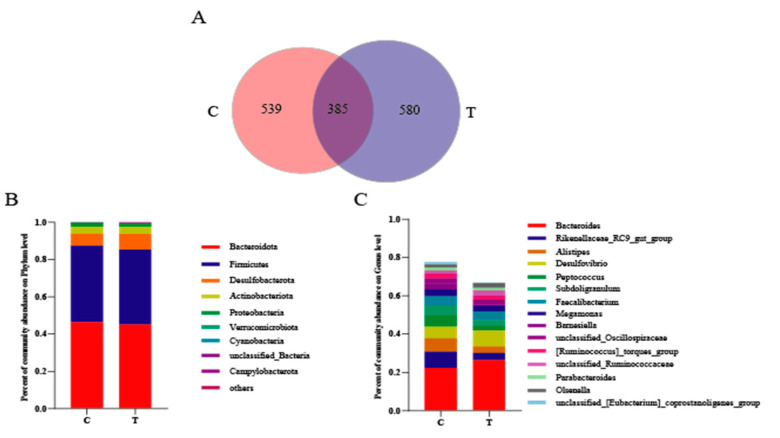
Venn diagram of OTUs and dominant cecum bacterial taxa analysis between the control group (**C**) and 3.2% CA group (T). (**A**) The Venn diagram summarizing the shared and unique OTUs in cecal microbiota. (**B**,**C**) Percent of community abundance of the cecal microbial community at the phylum and genus level.

Figure 2A shows the common and unique features between the two groups. A total of 924 and 965 OTUs were clustered in the control and 3.2% CA group, respectively, and the 3.2% CA group showed more unique OTUs (580) than the control (539). In regard to the dominant phyla and genera of the bacteria in the cecum, the data showed that Bacteroidota, Firmicutes, Desulfobacterota, Actinobacteriota, and Proteobacteria were the most abundant phyla (Figure 2B), while *Bacteroides*, *Alistipes*, *Desulfovibrio*, *Peptococcus*, *Subdoligranulum*, *Faecalibacterium*, *Megamonas,* and *Barnesiella* were the most abundant bacteria (Figure 2C).

**Figure 3:**
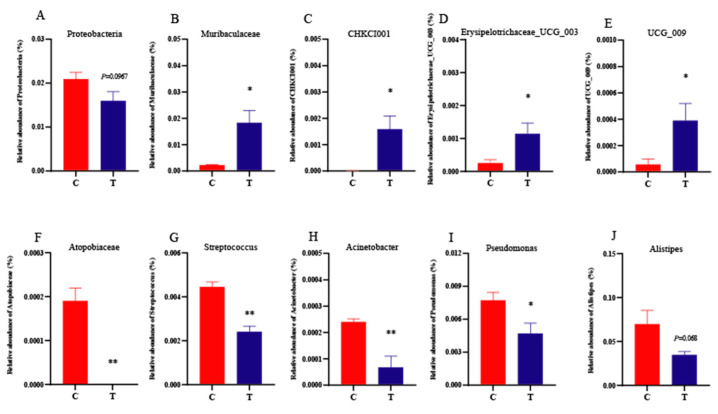
Effects of dietary 3.2% CA supplementation on cecal contents’ specific microbiota of geese at 70 d of age. (**A**–**J**) The relative abundance of cecal microbiota at the phylum and genus level with significant variations. C = the control group, T = the 3.2% CA group. * *p* < 0.05, ** *p* < 0.01.

Moreover, the Proteobacteria in the 3.2% CA group showed a significant decreasing trend (*p* = 0.0967, Figure 3A) at the phylum level. At the genus level, 3.2% CA supplementation significantly increased the abundance of *Muribaculaceae*, *CHKCI001*, *Erysipelotrichaceae_UCG_003*, and *UCG_009* (*p* < 0.05, Figure 3B–E), whereas some potential pathogenic bacteria, including *Pseudomonas* (*p* < 0.05), *Atopobiaceae* (*p* < 0.01), *Streptococcus* (*p* < 0.01), *Acinetobacter* (*p* < 0.01), and *Alistipes* (*p* < 0.1), were decreased due the addition of CA (Figure 3F–J).

## 4. Discussion

Supplemental acidifiers for disease prevention and growth promotion are an efficient and price competitive nutritional regulation method. The current research found that 2.4% and 3.2% CA supplementation exerted a favorable growth-promoting function, and this result was in line with Abdel-Fattah et al. [10], who previously demonstrated that the addition of 2% or 3% CA in broiler diets markedly improved FBW and F/G. A.Nouri et al. [19] also demonstrated that the ADFI and BW of broilers were improved by 1.5% and 3% CA supplementation. And the improvement in the F/G was also verified by some other studies [20,21]. The acidifying effect of CA on diets could have been responsible for the appetite stimulation and activation of digestive enzymes, and eventually led to the improvement of performance data, which was further verified by the decrease in the pH value and acid-binding capacity of treatment diets. Furthermore, Nourmohammadi et al. [22] illustrated that broilers fed 6% CA diets resulted in adverse effects on ADFI and ADG compared with the control group and 3% CA group, which means that excess citric acid might adversely affect growth performance. Similarly, Fikry et al. [6] discovered that adding 0.5% to 2% CA to Japanese quails’ diets improved FBW and ADG, but that growth performance increased initially and then decreased with increasing levels of CA, with the best performance achieved at 1%. Esmaeilipour et al. [23] also found 4% CA supplementation in diets adversely affected the ADG and ADFI of broilers. Our data revealed that the FBW, ADG, and ADFI of the group fed the 4% CA supplemented diet did not improve further or were even repressed, which meant that the inclusion of 4% CA might have been close to the maximum acid-resistant capacity of growing geese, thus severely affecting the growth performance and the efficiency of nutrient absorption. Therefore, the diet should be scientifically supplemented with appropriate levels of CA for the purpose of growth promotion.

In the present study, the improvement in performance due to the addition of 3.2% CA was further verified by the carcass traits, the increase in the TMP, and the decrease in the AFP and SFP, indicating that CA might be beneficial to the increase in net available energy for protein synthesis by improving the oxidation of fatty acids or restraining the biosynthesis of fatty acids [24]. Consistent with our findings, Haq et al. [25] found that with the increase in CA addition to 1.5%, the DP significantly increased, while the ABP significantly decreased in ducklings. Moreover, a significant reduction in ABP was also found in 2% or 3% CA supplemented diets by Elnagar et al. [11]. The proventriculus–gizzard indices and liver indices of broilers were significantly decreased by 0.25% CA [26]; however, some results reported that CA supplementation in broiler and quail diets did not significantly affect carcass traits [6,27]. This divergence may be because of the differences in animal species, genders, age, supplemental dosages, and the composition of ingredients.

Several blood metabolites can be used to determine the animals’ physiological status and overall health. Our results found that plasma ALB remained unchanged, whereas GLO and TP first increased and then decreased as dietary CA increased. The geese fed a 4% CA diet had significantly lower GLO and TP than the group fed a 1.6% or 2.4% CA diet. Similarly, previous studies found that broilers fed a diet containing 3% acidifier showed higher blood GLO and immune organ weight than the control group [10]. Moreover, Ghazalah et al. [28] found that the TP and GLO levels of broilers were significantly increased by the addition of 1% to 2% CA, whereas no significant difference was observed in broilers who received an addition of 3% CA. Furthermore, consistent with some previous studies [6,11,29], the decreased plasma Urea and UA levels, which are the main end products of N metabolism, implied that the CA supplementation in geese diets may be beneficial to the efficiency of amino acid utilization and renal function. In addition, previous studies have shown that high plasma UA levels were positively correlated with long-term purine metabolism disorder, which eventually led to gout [30]. Furthermore, in line with our findings, Elnagar et al. [11] reported that ducklings fed a diet containing 2% or 3% CA showed lower CREA and Urea levels. Fikry et al. [6] found that the increase in the amount of CA added, from 0.5% to 1.5%, results in a reduction in plasma Urea levels. Reda et al. [29] demonstrated that 0.5% to 2% CA supplemented diet reduced quails’ Urea levels, while no significant difference was observed for their CREA levels. Overall, these results imply that the immune status and amino acid utilization efficiency of growing geese could be enhanced by CA supplementation.

IgA, IgG, and IgM are the main antibodies mediating humoral immunity, and increased immunoglobulins have been associated with improved immune function [31]. T-AOC, GSH-Px, SOD, and CAT are all considered vital indicators of antioxidant status in the body, which can prevent oxidative stress by scavenging free radicals such as reactive oxygen species. MDA has been recognized as a biomarker of lipid peroxidation, which indirectly reflects the degree of cellular damage [32]. Our results found the levels of plasma GSH-Px were increased in the 2.4% and 3.2 CA groups, suggesting that the antioxidant system was improved to sustain oxidative stability. Our findings are partly consistent with those of Elnagar et al. [11], who found that CA increased the plasma T-AOC, GSH-Px, and SOD activities of ducklings; however, Mustafa et al. [33] demonstrated that broilers fed a diet containing organic acids had higher plasma IgG levels. Fikry et al. [6] demonstrated that quails treated with 5 or 10 g/kg CA supplementation showed significantly higher IgG levels, while IgA levels were significantly reduced with CA addition (up to 20 g/kg). This difference was probably caused by different species or ages. Furthermore, some other organic acids also showed an antioxidant capacity in poultry diets [34,35], but their mechanism needs to be further researched.

The secretion and metabolism of hormones are essential for birds’ growth, development, and reproduction. GHs are peptide hormones secreted by the pituitary gland, which plays a vital role in promoting metabolism and maintaining normal development. IGF-1 is a polypeptide similar in structure and function to insulin, which exerts control over the cell growth cycle, maturation, differentiation, proliferation, and protein anabolism [36,37]. In this study, supplementing diets with 3.2% CA noticeably increased the level of IGF-1, which might account for the improvement of growth performance observed in the 3.2% CA group.

The gut pH value is directly associated with gastric acid secretion and digestive enzyme activity, thus impacting the absorption of nutrients [38]. In the present study, we found that dietary the addition of CA significantly decreased the pH value and acid-binding capacity of diets; however, only the jejunum content’s pH value observed a significant decrease due to the addition of CA. Similar to our previous findings [14], the diets containing CA only significantly reduced the jejunal content’s pH value. Nourmohammadi et al. [22] also reported that the addition of 3% CA only significantly reduced the jejunal content’s pH value, while no significant differences were found in the content pH value of other segments of the GIT. However, some studies found that the addition of an acidifier to water significantly reduced the pH value of all segments of the GIT [39,40]. This difference indicates that the acidifier’s effect on gut pH value regulation differs based on species, tract segments, dosages, and the form of the acidifier used.

The small intestine plays an essential role in the digestion and absorption of nutrients, and small intestine morphology indicators (VH, CD, and VH/CD) are directly associated with the digestive capacity and intestinal health of animals. In this study, geese from the 3.2% CA group showed the highest duodenum and jejunum VH/CD, indicating that the intestinal absorption capacity was improved by CA. These results coincide with those of Nourmohammadi et al. [7], who reported that 3% and 6% CA supplementation in broilers significantly increased duodenum VH/CD. The positive effect of CA on the intestinal development of poultry has also been reported by Khosravinia et al. [41]. The increase in VH/CD may be related to the antibacterial ability of CA as an organic acidifier, which may be beneficial as it disrupts bacterial layers and interferes with the bacterial metabolism of many pathogenic bacteria, ultimately preventing intestinal damage [42]. Noteworthily, excess CA (4%) supplementation exerted no further beneficial effects on small intestinal morphology in the VH/CD of the duodenum and jejunum compared with the 3.2% CA group, which may be directly associated with the decreased feed consumption.

The cecum is the main place for microbial fermentation in poultry. As shown by the beta diversity, the dietary supplementation of 3.2% CA had a significant effect on cecum microbiota communities. This can be presumably explained by the reduced pH value in the gut, which is consistent with several findings on CA [6,10,14]. Clustering results showed that Firmicutes and Bacteroidota are the top two dominant bacterial phyla in geese ceca, which is consistent with the findings of Fang et al. [43]. Differential analysis results showed that 3.2% CA supplementation in diets increased the relative abundance of several probiotics, including *Muribaculaceae*, *CHKCI001*, *Erysipelotrichaceae_UCG_003*, and *UCG_009*, which contributes to a reduction in damage caused by intestinal disease. *Muribaculaceae* is a beneficial bacterium in the intestine, belonging to the genus Mycobacterium. Increased *Muribaculaceae* was considered to be directly associated with a prolonged lifespan and improved anti-inflammatory functions in mammals [44]. *Erysipelotrichaceae_UCG_003* is known as a key butyrate-producing member, which is positively associated with the antioxidant and anti-inflammatory capabilities in birds [45,46]. Additionally, *CHKCI001* and *UCG_009* are generally regarded as growth-promoting bacteria. Zheng et al. [47] reported that *CHKCI001* was positively associated with growth performance in geese. *UCG_009* can promote the hydrolyzation of proteins into polypeptides and amino acids [48]. Meanwhile, several pathogens such as Proteobacteria, *Atopobiaceae*, *Streptococcus*, *Acinetobacter*, *Pseudomonas*, and *Alistipes* were observed to be reduced by CA supplementation in our study. Proteobacteria is the major phylum of Gram-negative bacteria, and the decreased abundance of Proteobacteria can alleviate intestinal damage and maintain organ health [49]. *Atopobiaceae*, *Streptococcus*, and *Pseudomonas* are common opportunistic pathogens in poultry, which are highly associated with inflammation [50]. *Acinetobacter* is reported to be harmful to broiler’s respiratory system and to increase the mortality rate during broiler breeding [51]. Cobo et al. [52] pointed that *Alistipes* is highly relevant to chronic intestinal inflammation, and other studies demonstrated that *Alistipes* is positively correlated with cancer progression and mental disorders [53]. Considering the improvement in growth performance, carcass traits, antioxidant status, and small intestinal morphology in 3.2% CA supplemented geese, we can surmise that the addition of CA could stimulate intestinal development and maintain the health of the host’s gut by facilitating the abundance of several probiotics and reducing the abundance of several pathogens.

## 5. Conclusions

Our results revealed that dietary CA supplementation had a favorable effect on performance, antioxidant status, small intestinal development, and cecum microbiota in growing geese. Under our experimental conditions, an inclusion of 3.2% CA in growing geese’s diets is recommended.

## Figures and Tables

**Table 1:** Ingredients and chemical composition of basal diet on a feed basis.

Items	%
Ingredients	
Maize	54.00
Soybean meal	20.50
Wheat bran	15.00
Paddy rice	6.80
Limestone	1.10
Calcium hydrogen phosphate	1.50
Sodium chloride	0.40
DL-Methionine	0.30
Choline chloride	0.10
Mineral and vitamin premix ^1^	0.30
Total	100
Calculated nutrient levels	
Metabolizable energy (MJ/kg)	12.06
Crude protein	16.22
Calcium	0.80
Total phosphorus	0.65
Lysine	0.90
Methionine	0.45
Analyzed values	
Crude protein	16.00
Ether extract	2.80
Lysine	0.92
Methionine	0.40

^1^ Supplied by premix for per kg of diet: Cu (CuSO_4_·5H_2_O), 8 mg; Fe (FeSO_4_·H_2_O), 96 mg; Zn (ZnSO_4_·H_2_O), 80 mg; Mn (MnSO_4_·H_2_O), 100 mg; Se (Na_2_SeO_3_), 0.3 mg; I (KI), 0.4 mg; pantothenic acid, 10 mg; nicotinic acid, 50 mg; folic acid, 0.5 mg; biotin, 0.15 mg; vitamin A, 6000 IU; vitamin D_3_, 1500 IU; vitamin E, 10 IU; vitamin K_3_, 2.4 mg; vitamin B_1_, 1.5 mg; vitamin B_2_, 5 mg; vitamin B_6_, 3 mg; vitamin B_12_, 0.02 mg.

**Table 2:** Effects of citric acid on pH value and acid-binding capacity of diets.

Items	Citric Acid Level %						SEM	*p*-Value
	0	0.8	1.6	2.4	3.2	4		
pH value	6.43 ^a^	5.90 ^b^	5.53 ^c^	5.28 ^d^	5.11 ^e^	5.08 ^f^	0.116	0.0001
Acid-binding capacity (mL/100 g feed)	64.7 ^a^	60.8 ^b^	58.6 ^b^	48.1 ^c^	35.2 ^d^	25.3 ^e^	3.477	0.0001

^a, b, c, d, e, f^ Means in the same row with different superscripts indicate a significant difference (*p* < 0.05).

**Table 3:** Effects of citric acid on production performance of growing geese.

Items	Citric Acid Level %						SEM	*p*-Value
	0	0.8	1.6	2.4	3.2	4		
IBW (g/bird)	1295.8	1296.6	1298.6	1294.1	1295.6	1297.0	3.06	0.999
FBW (g/bird)	3012.8 ^b^	3019.0 ^b^	3060.6 ^b^	3171.7a ^b^	3241.9 ^a^	3015.9 ^b^	21.91	0.001
ADG (g/bird per day)	40.88 ^c^	40.94 ^c^	42.06 ^bc^	44.70 ^ab^	46.45 ^a^	40.88 ^c^	0.52	0.000
ADFI (g/bird per day)	196.69 ^b^	199.95 ^b^	200.80 ^ab^	213.15 ^a^	212.71 ^a^	193.53 ^b^	1.83	0.000
F/G (g/g)	4.82	4.89	4.77	4.77	4.58	4.73	0.03	0.083

^a, b, c^ Means in the same row with different superscripts indicate a significant difference (*p* < 0.05). Abbreviations: IBW, initial body weight; FBW, final body weight; ADG, average daily gain; ADFI, average daily feed intake; F/G, feed/gain.

**Table 4:** Effects of citric acid on carcass traits of growing geese.

Items	Citric Acid Level %						SEM	*p*-Value
	0	0.8	1.6	2.4	3.2	4		
DP	89.28	89.32	88.41	87.84	88.82	88.52	0.24	0.500
EP	75.60	75.79	74.64	75.16	75.60	75.33	0.36	0.963
BMP	8.47	8.01	8.25	9.08	8.46	8.75	0.24	0.846
TMP	10.92 ^b^	12.89 ^ab^	13.30 ^a^	12.54 ^ab^	12.05 ^ab^	13.35 ^a^	0.24	0.045
SFP	20.61 ^a^	18.96 ^ab^	18.08 ^ab^	18.30 ^ab^	16.97 ^ab^	16.50 ^b^	0.42	0.025
AFP	4.51 ^a^	3.10 ^ab^	3.16 ^ab^	2.75 ^b^	3.31 ^ab^	2.23 ^b^	0.20	0.004
HP	0.67	0.68	0.61	0.68	0.68	0.66	0.01	0.832
LP	2.53	2.50	2.71	3.19	2.68	3.08	0.10	0.251
PGP	3.03	2.94	2.99	3.12	2.80	2.90	0.09	0.964

^a, b^ Means in the same row with different superscripts indicate a significant difference (*p* < 0.05). Abbreviations: DP, dressing percentage; EP, eviscerated percentage; BMP, breast muscle percentage; TMP, thigh muscle percentage; SFP, subcutaneous fat with skin percentage; AFP, abdominal fat percentage; HP, heart percentage; LP, liver percentage; PGP, proventriculus–gizzard percentage.

**Table 5:** Effects of citric acid on plasma constituents of growing geese.

Items	Citric Acid Level %						SEM	*p*-Value
	0	0.8	1.6	2.4	3.2	4		
Metabolites								
TP (g/L)	43.10 ^ab^	44.34 ^ab^	45.40 ^ab^	45.90 ^a^	44.50 ^ab^	41.68 ^b^	0.430	0.037
ALB (g/L)	12.08	12.24	12.38	12.46	12.38	11.84	0.096	0.447
GLO (g/L)	31.02 ^ab^	32.10 ^ab^	33.02 ^ab^	33.44 ^a^	32.12 ^ab^	29.84 ^b^	0.361	0.030
Urea (mmol/L)	0.33 ^a^	0.17 ^b^	0.22 ^ab^	0.25 ^ab^	0.17 ^b^	0.21 ^ab^	0.015	0.012
CREA (μmol/L)	15.50	13.50	16.00	15.44	15.16	15.80	0.287	0.136
UA (μmol/L)	224.4 ^a^	206.4 ^a^	140.5 ^b^	180.6 ^ab^	180.5 ^ab^	173.0 ^ab^	6.720	0.002
Immune indices								
IgA (g/L)	3.12	3.26	3.25	3.11	3.07	2.98	0.070	0.885
IgG (g/L)	8.16	10.25	9.39	9.30	9.30	9.08	0.224	0.183
IgM (g/L)	0.85	0.86	0.86	0.87	0.85	0.79	0.022	0.913
Antioxidant capacity								
T-AOC (U/mL)	0.75	0.75	0.75	0.76	0.78	0.70	0.008	0.091
GSH-Px (U/mL)	569.6 ^ab^	537.1 ^ab^	540.8 ^ab^	616.8 ^ab^	653.2 ^a^	529.3 ^b^	13.37	0.019
SOD (U/mL)	776.6	790.7	816.5	824.4	804.2	768.6	16.96	0.937
MDA (nmol/mL)	5.33	5.47	5.51	5.28	5.13	5.25	0.136	0.977
CAT (μmol/mL)	0.79	0.79	0.77	0.80	0.79	0.64	0.038	0.887
Hormones								
GH (ng/mL)	2.13	2.02	2.10	2.12	2.45	2.02	0.065	0.410
IGF-1 (ng/mL)	58.20 ^b^	74.05 ^ab^	68.93 ^ab^	70.58 ^ab^	84.00 ^a^	62.92 ^ab^	2.570	0.039

^a, b^ Means in the same row with different superscripts indicate a significant difference (*p* < 0.05). Abbreviations: TP, total protein; ALB, albumin; GLO, globulin; CREA, creatinine; UA, uric acid; T-AOC, total antioxidant capacity; GSH-Px, glutathione peroxidase; SOD, superoxide dismutase; MDA, malondialdehyde; CAT, catalase; GH, growth hormone; IGF-1, insulin-like growth factor 1.

**Table 6:** Effects of citric acid on small intestinal pH and morphology of growing geese.

Items	Citric Acid Level %						SEM	*p*-Value
	0	0.8	1.6	2.4	3.2	4		
Duodenum								
pH	6.47	6.29	6.34	6.41	6.38	6.15	0.032	0.092
VH (μm)	902.0	882.7	910.1	960.7	920.3	988.2	13.25	0.173
CD (μm)	140.4	126.6	133.2	129.2	119.0	136.0	2.556	0.218
VH/CD	6.46	7.01	6.83	7.71	7.79	7.31	0.153	0.087
Jejunum								
pH	6.89	6.91	6.84	6.65	6.54	6.42	0.058	0.087
VH (μm)	1079.1	1154.4	1187.1	1207.7	1196.8	1264.6	24.77	0.397
CD (μm)	101.3	104.3	101.5	102.7	95.5	104.0	1.400	0.521
VH/CD	10.50	11.06	11.71	12.32	12.69	12.21	0.251	0.080
Ileum								
pH	6.82	6.72	6.75	6.90	6.97	6.96	0.063	0.829
VH (μm)	772.9	732.2	847.4	843.4	816.1	784.0	16.81	0.349
CD (μm)	110.8	102.4	116.6	113.3	111.4	106.3	2.351	0.592
VH/CD	7.03	7.16	7.16	7.48	7.43	7.46	0.148	0.937

## Data Availability

All relevant data are within the manuscript.
